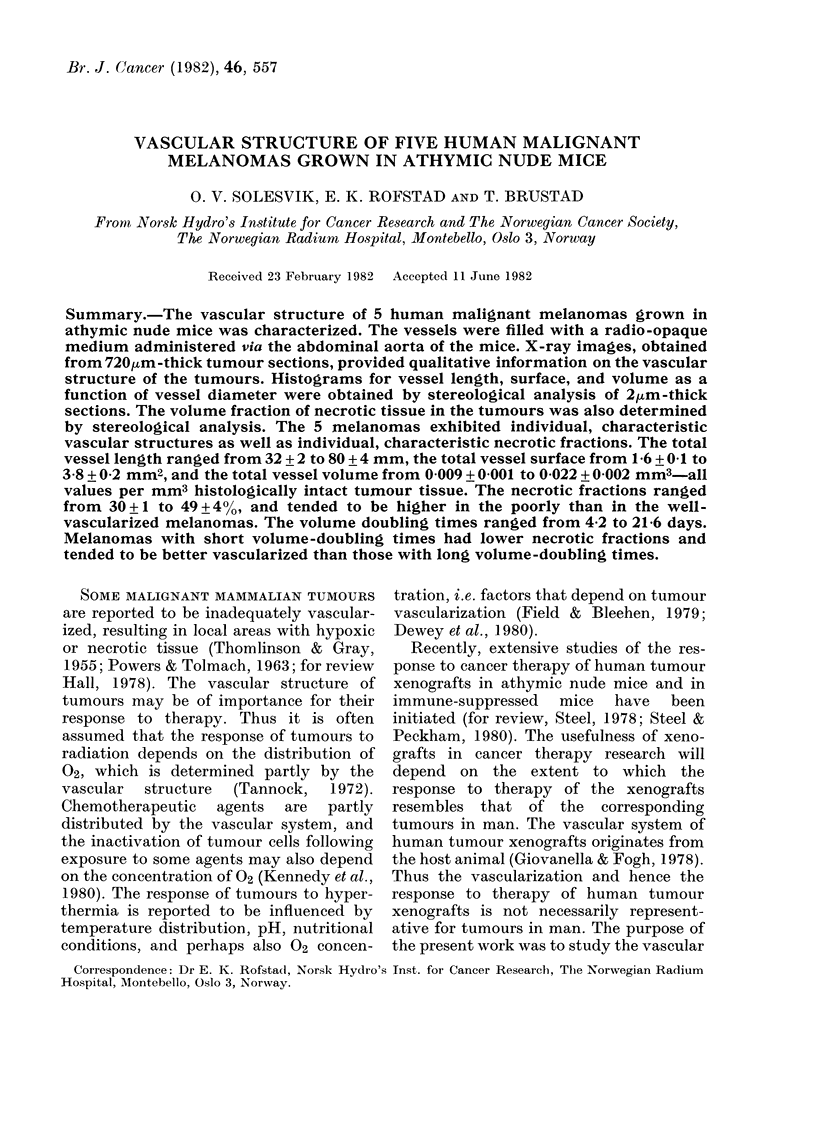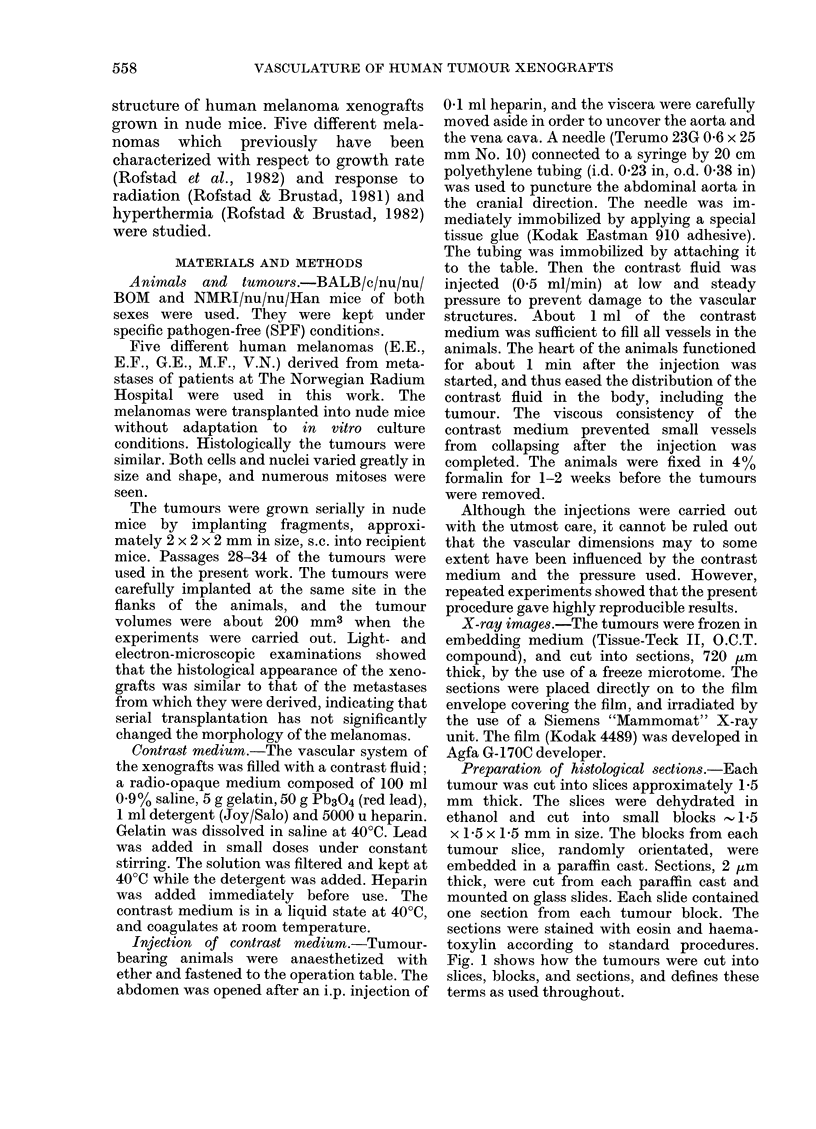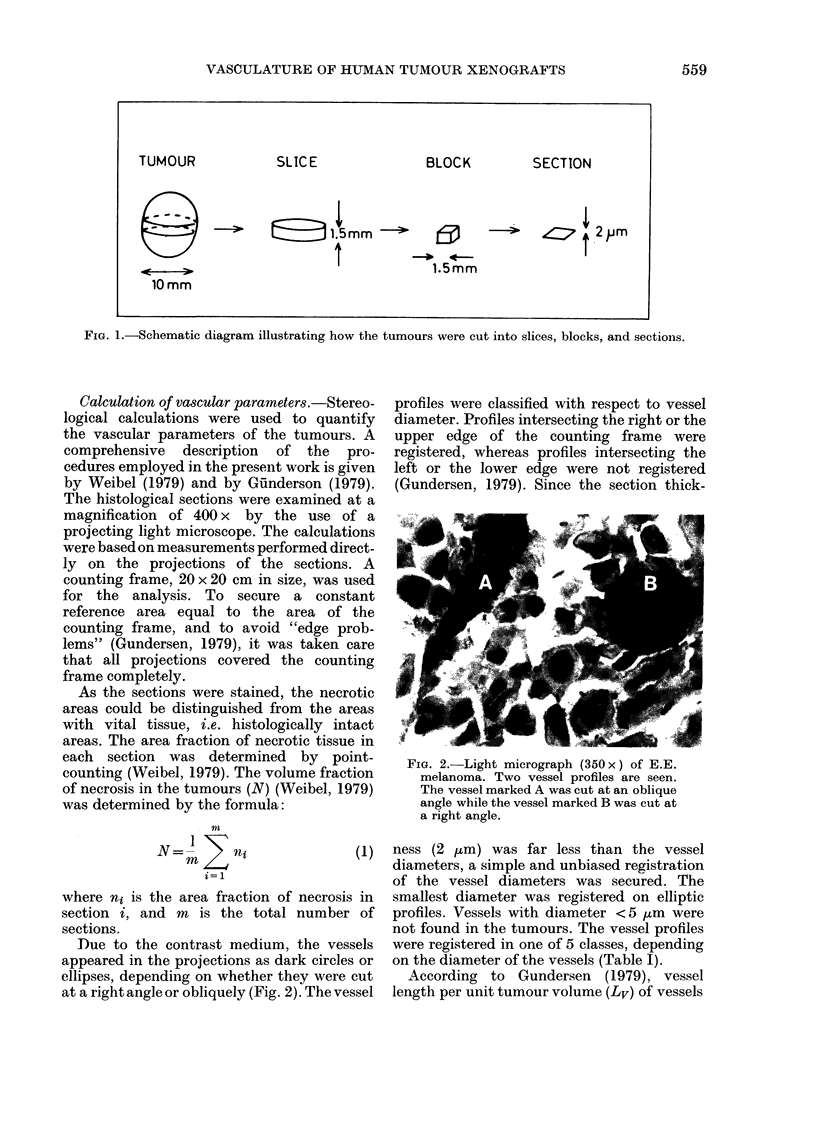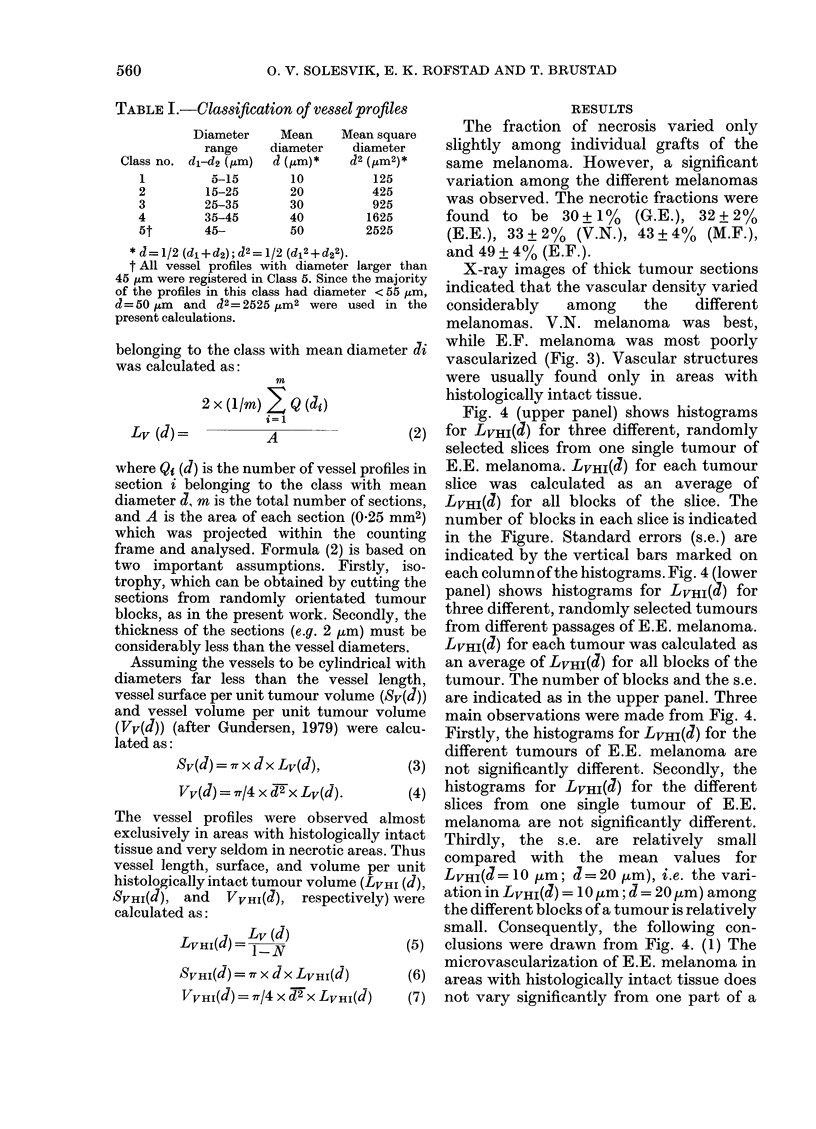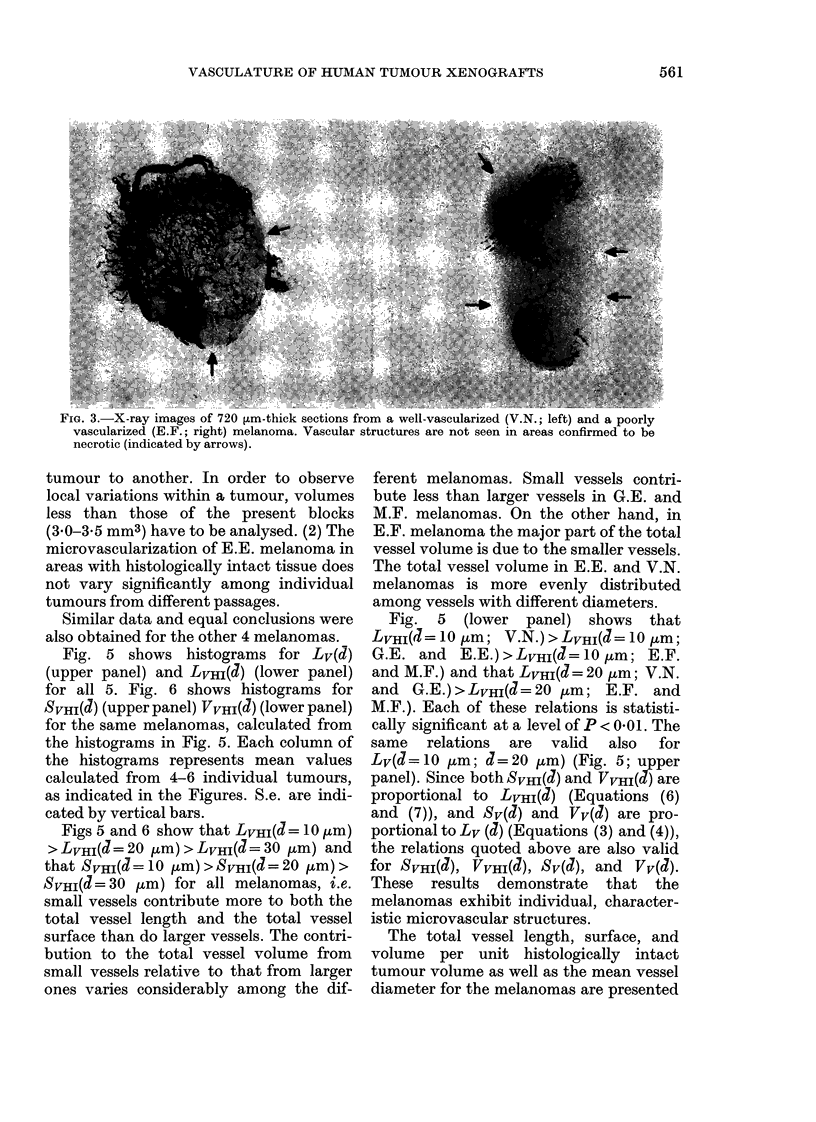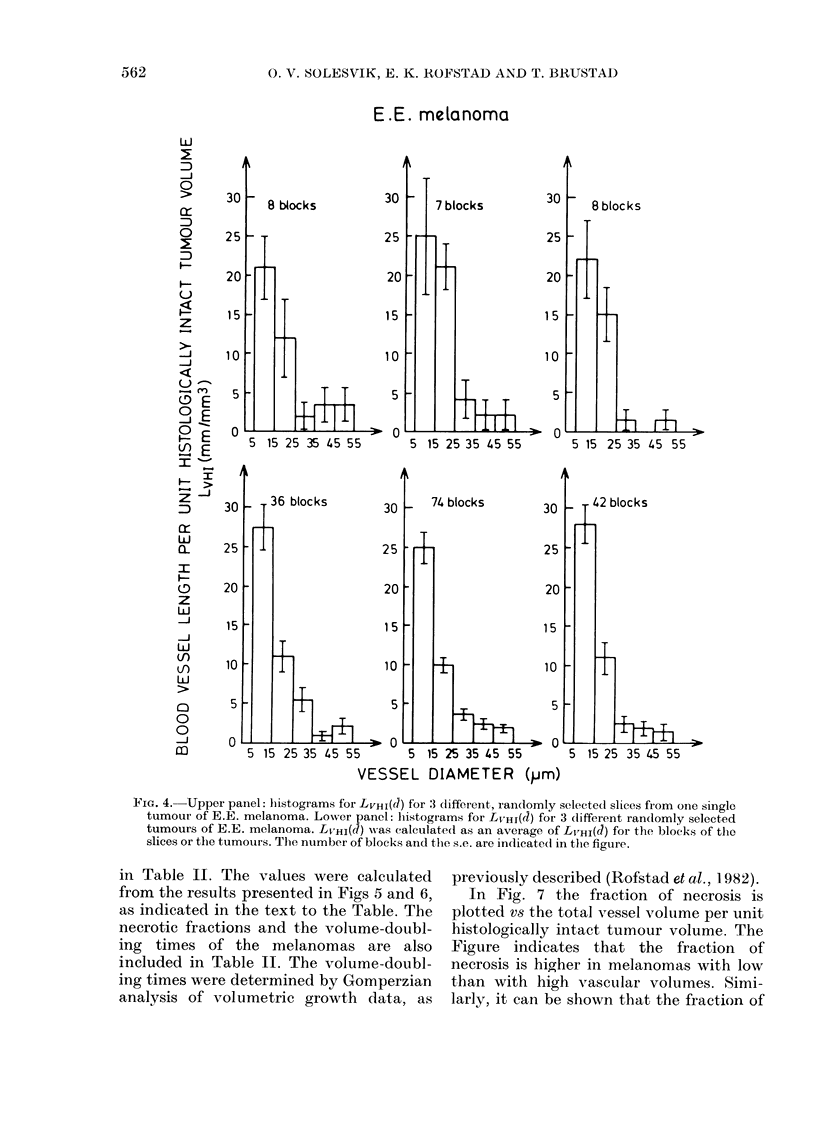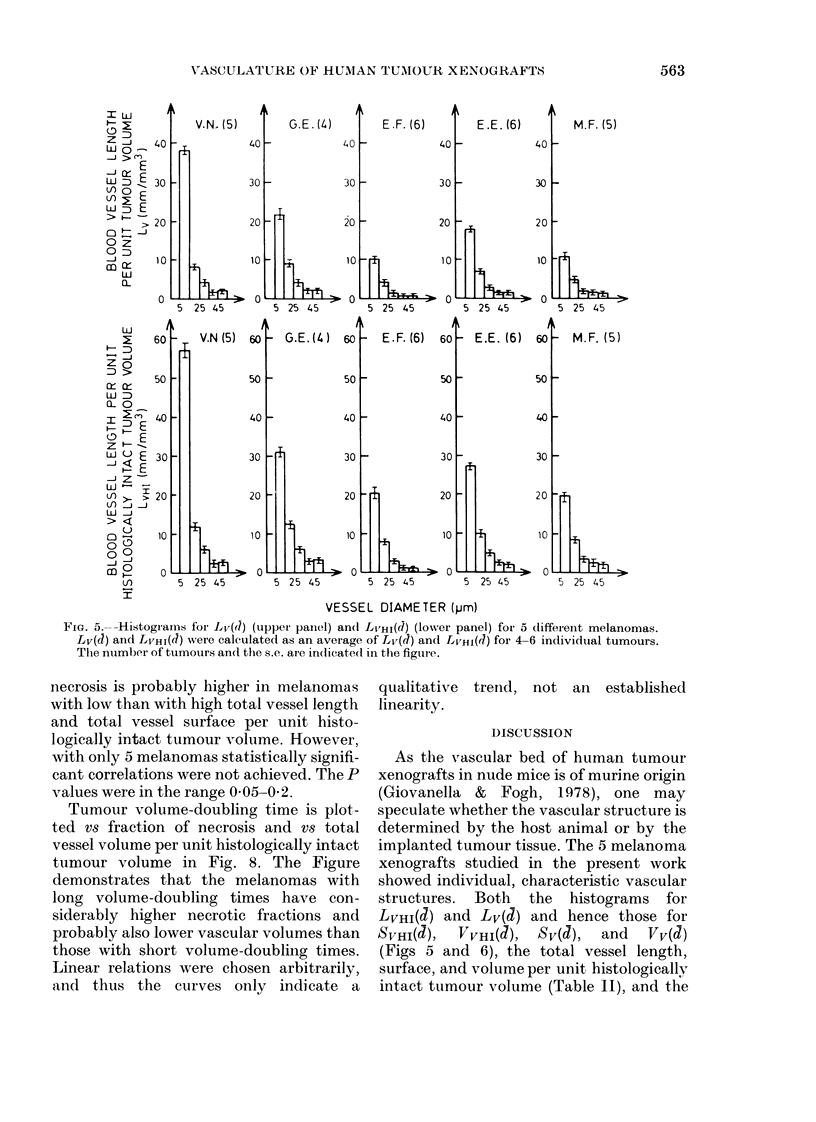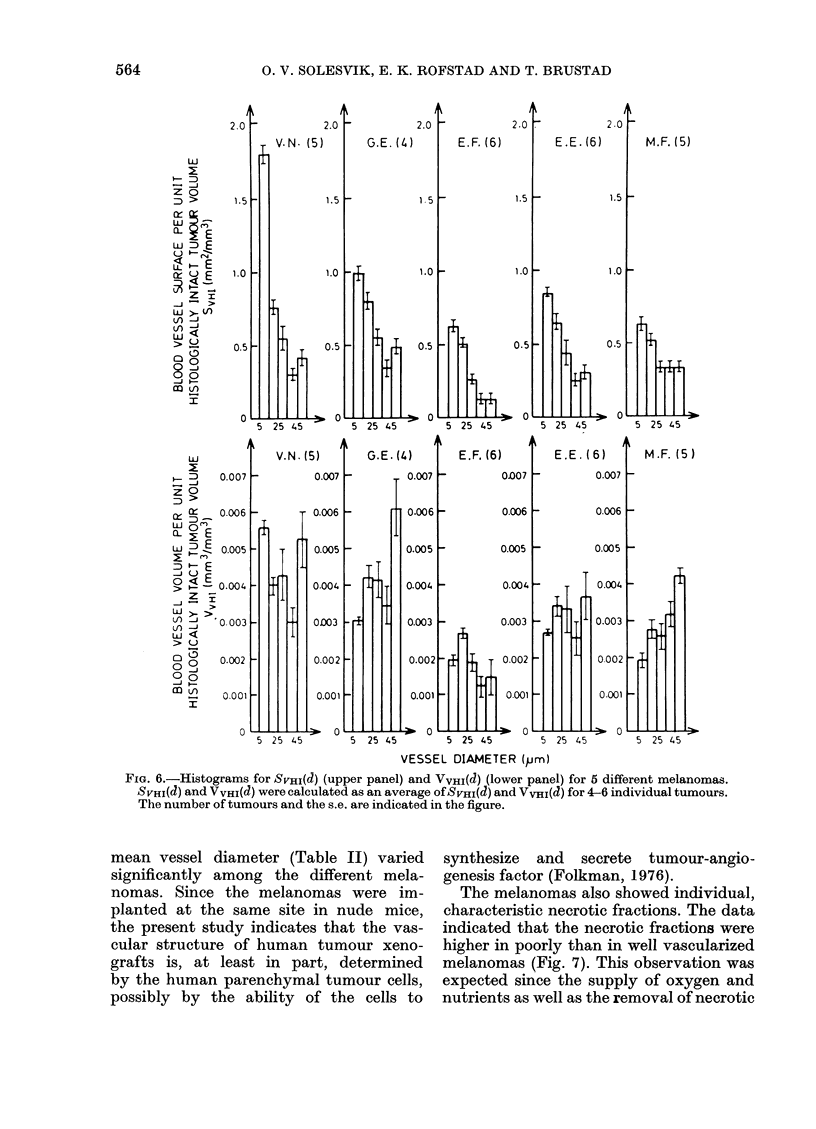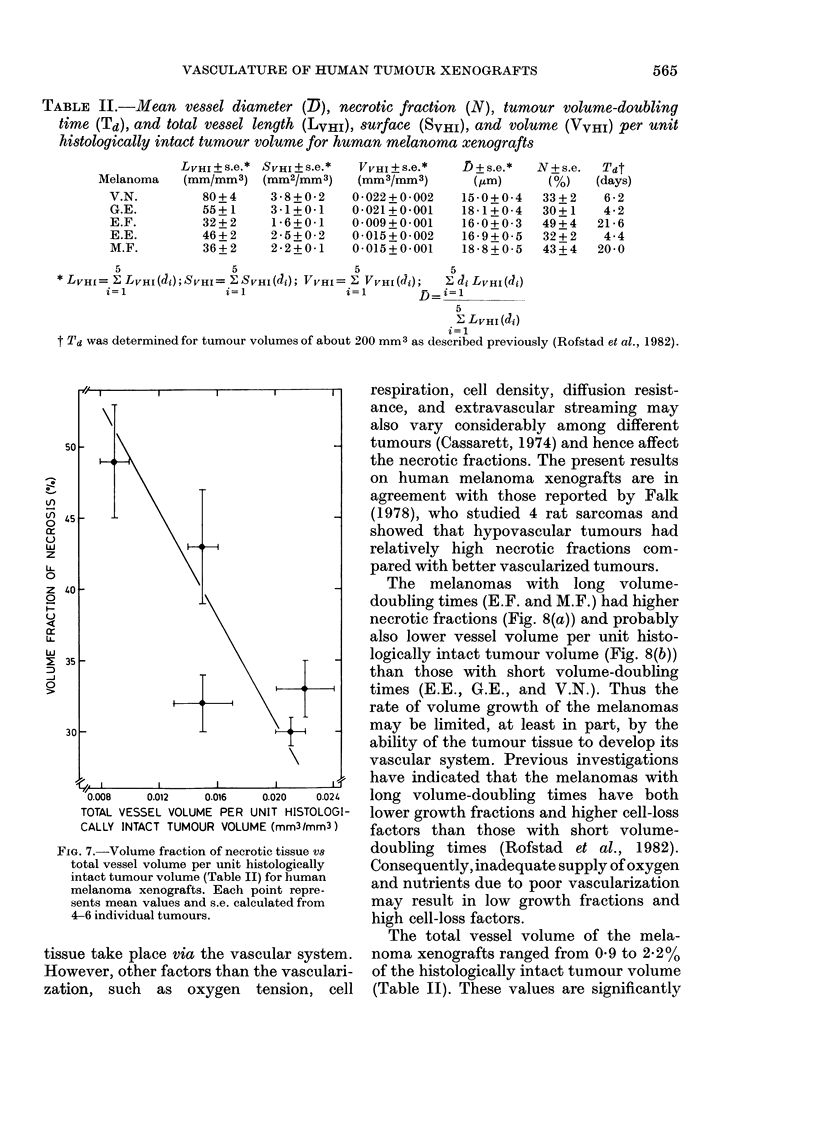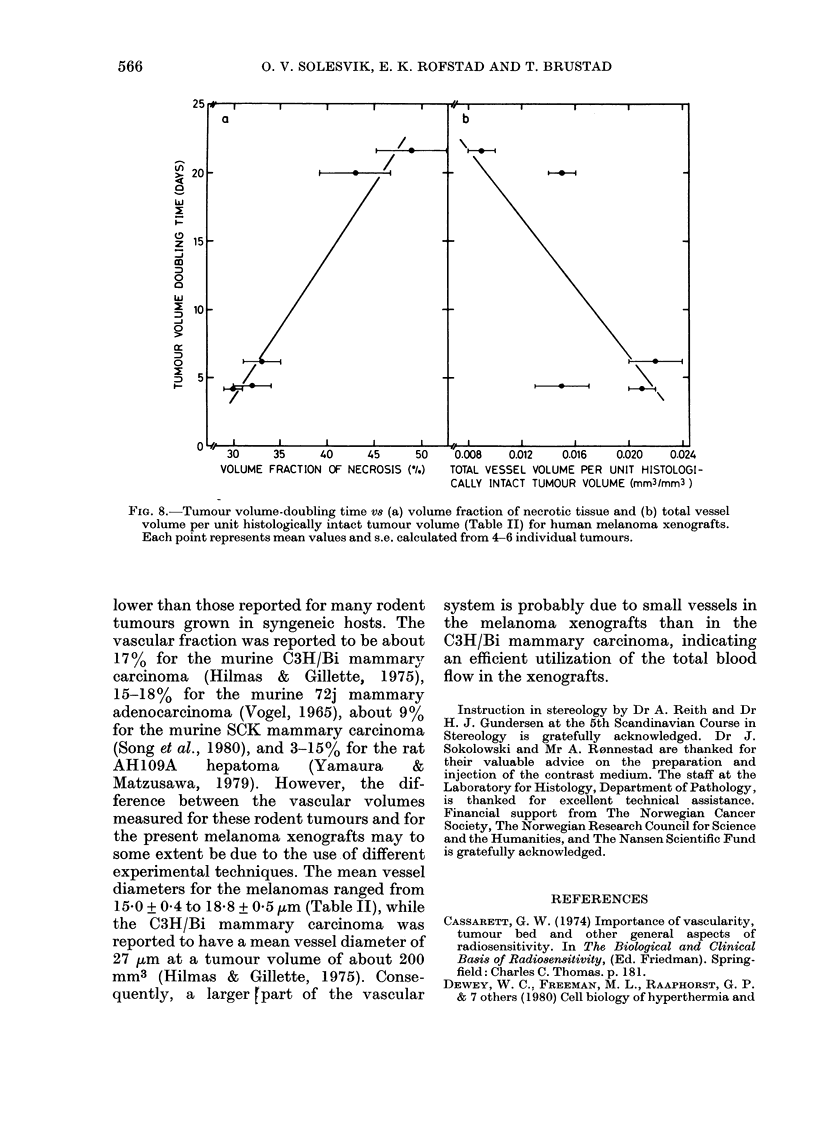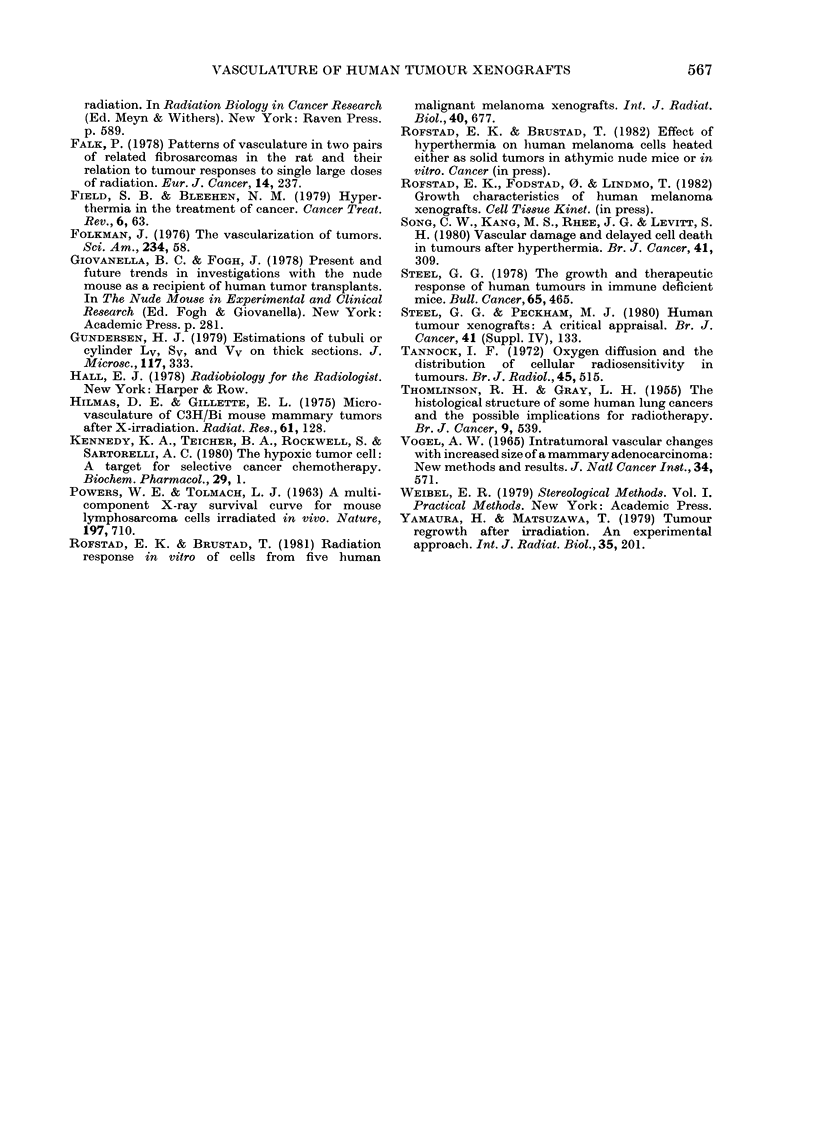# Vascular structure of five human malignant melanomas grown in athymic nude mice.

**DOI:** 10.1038/bjc.1982.240

**Published:** 1982-10

**Authors:** O. V. Solesvik, E. K. Rofstad, T. Brustad

## Abstract

**Images:**


*Br. J. Cancer* (1982), **46**, 557

### VASCULAR STRUCTURE OF FIVE HUMAN MALIGNANT MELANOMAS GROWN IN ATHYMIC NUDE MICE

O. V. SOLESVIK, E. K. ROFSTAD AND T. BRUSTAD

*From Norsk Hydro's Institute for Cancer Research and The Norwegian Cancer Society, The Norwegian Radium Hospital, Montebello, Oslo 3, Norway*

Received 23 February 1982 Accepted 11 June 1982

**Summary.—The vascular structure of 5 human malignant melanomas grown in athymic nude mice was characterized. The vessels were filled with a radio-opaque medium administered *via* the abdominal aorta of the mice. X-ray images, obtained from 720$\mu$m-thick tumour sections, provided qualitative information on the vascular structure of the tumours. Histograms for vessel length, surface, and volume as a function of vessel diameter were obtained by stereological analysis of 2$\mu$m-thick sections. The volume fraction of necrotic tissue in the tumours was also determined by stereological analysis. The 5 melanomas exhibited individual, characteristic vascular structures as well as individual, characteristic necrotic fractions. The total vessel length ranged from $32 \pm 2$ to $80 \pm 4$ mm, the total vessel surface from $1{\cdot}6 \pm 0{\cdot}1$ to $3{\cdot}8 \pm 0{\cdot}2$ mm$^2$, and the total vessel volume from $0{\cdot}009 \pm 0{\cdot}001$ to $0{\cdot}022 \pm 0{\cdot}002$ mm$^3$—all values per mm$^3$ histologically intact tumour tissue. The necrotic fractions ranged from $30 \pm 1$ to $49 \pm 4\%$, and tended to be higher in the poorly than in the well-vascularized melanomas. The volume doubling times ranged from 4·2 to 21·6 days. Melanomas with short volume-doubling times had lower necrotic fractions and tended to be better vascularized than those with long volume-doubling times.**

Some malignant mammalian tumours are reported to be inadequately vascularized, resulting in local areas with hypoxic or necrotic tissue (Thomlinson & Gray, 1955; Powers & Tolmach, 1963; for review Hall, 1978). The vascular structure of tumours may be of importance for their response to therapy. Thus it is often assumed that the response of tumours to radiation depends on the distribution of $O_2$, which is determined partly by the vascular structure (Tannock, 1972). Chemotherapeutic agents are partly distributed by the vascular system, and the inactivation of tumour cells following exposure to some agents may also depend on the concentration of $O_2$ (Kennedy *et al.*, 1980). The response of tumours to hyperthermia is reported to be influenced by temperature distribution, pH, nutritional conditions, and perhaps also $O_2$ concentration, *i.e.* factors that depend on tumour vascularization (Field & Bleehen, 1979; Dewey *et al.*, 1980).

Recently, extensive studies of the response to cancer therapy of human tumour xenografts in athymic nude mice and in immune-suppressed mice have been initiated (for review, Steel, 1978; Steel & Peckham, 1980). The usefulness of xenografts in cancer therapy research will depend on the extent to which the response to therapy of the xenografts resembles that of the corresponding tumours in man. The vascular system of human tumour xenografts originates from the host animal (Giovanella & Fogh, 1978). Thus the vascularization and hence the response to therapy of human tumour xenografts is not necessarily representative for tumours in man. The purpose of the present work was to study the vascular

Correspondence: Dr E. K. Rofstad, Norsk Hydro's Inst. for Cancer Research, The Norwegian Radium Hospital, Montebello, Oslo 3, Norway.

structure of human melanoma xenografts grown in nude mice. Five different melanomas which previously have been characterized with respect to growth rate (Rofstad *et al.*, 1982) and response to radiation (Rofstad & Brustad, 1981) and hyperthermia (Rofstad & Brustad, 1982) were studied.

### MATERIALS AND METHODS

*Animals and tumours.*—BALB/c/nu/nu/BOM and NMRI/nu/nu/Han mice of both sexes were used. They were kept under specific pathogen-free (SPF) conditions.

Five different human melanomas (E.E., E.F., G.E., M.F., V.N.) derived from metastases of patients at The Norwegian Radium Hospital were used in this work. The melanomas were transplanted into nude mice without adaptation to *in vitro* culture conditions. Histologically the tumours were similar. Both cells and nuclei varied greatly in size and shape, and numerous mitoses were seen.

The tumours were grown serially in nude mice by implanting fragments, approximately $2 \times 2 \times 2$ mm in size, s.c. into recipient mice. Passages 28–34 of the tumours were used in the present work. The tumours were carefully implanted at the same site in the flanks of the animals, and the tumour volumes were about 200 $mm^3$ when the experiments were carried out. Light- and electron-microscopic examinations showed that the histological appearance of the xenografts was similar to that of the metastases from which they were derived, indicating that serial transplantation has not significantly changed the morphology of the melanomas.

*Contrast medium.*—The vascular system of the xenografts was filled with a contrast fluid; a radio-opaque medium composed of 100 ml 0·9% saline, 5 g gelatin, 50 g $Pb_3O_4$ (red lead), 1 ml detergent (Joy/Salo) and 5000 u heparin. Gelatin was dissolved in saline at 40°C. Lead was added in small doses under constant stirring. The solution was filtered and kept at 40°C while the detergent was added. Heparin was added immediately before use. The contrast medium is in a liquid state at 40°C, and coagulates at room temperature.

*Injection of contrast medium.*—Tumour-bearing animals were anaesthetized with ether and fastened to the operation table. The abdomen was opened after an i.p. injection of 0·1 ml heparin, and the viscera were carefully moved aside in order to uncover the aorta and the vena cava. A needle (Terumo 23G $0{\cdot}6 \times 25$ mm No. 10) connected to a syringe by 20 cm polyethylene tubing (i.d. 0·23 in, o.d. 0·38 in) was used to puncture the abdominal aorta in the cranial direction. The needle was immediately immobilized by applying a special tissue glue (Kodak Eastman 910 adhesive). The tubing was immobilized by attaching it to the table. Then the contrast fluid was injected (0·5 ml/min) at low and steady pressure to prevent damage to the vascular structures. About 1 ml of the contrast medium was sufficient to fill all vessels in the animals. The heart of the animals functioned for about 1 min after the injection was started, and thus eased the distribution of the contrast fluid in the body, including the tumour. The viscous consistency of the contrast medium prevented small vessels from collapsing after the injection was completed. The animals were fixed in 4% formalin for 1–2 weeks before the tumours were removed.

Although the injections were carried out with the utmost care, it cannot be ruled out that the vascular dimensions may to some extent have been influenced by the contrast medium and the pressure used. However, repeated experiments showed that the present procedure gave highly reproducible results.

*X-ray images.*—The tumours were frozen in embedding medium (Tissue-Teck II, O.C.T. compound), and cut into sections, 720 $\mu$m thick, by the use of a freeze microtome. The sections were placed directly on to the film envelope covering the film, and irradiated by the use of a Siemens "Mammomat" X-ray unit. The film (Kodak 4489) was developed in Agfa G-170C developer.

*Preparation of histological sections.*—Each tumour was cut into slices approximately 1·5 mm thick. The slices were dehydrated in ethanol and cut into small blocks $\sim 1{\cdot}5 \times 1{\cdot}5 \times 1{\cdot}5$ mm in size. The blocks from each tumour slice, randomly orientated, were embedded in a paraffin cast. Sections, 2 $\mu$m thick, were cut from each paraffin cast and mounted on glass slides. Each slide contained one section from each tumour block. The sections were stained with eosin and haematoxylin according to standard procedures. Fig. 1 shows how the tumours were cut into slices, blocks, and sections, and defines these terms as used throughout.

FIG. 1.—Schematic diagram illustrating how the tumours were cut into slices, blocks, and sections.

*Calculation of vascular parameters.*—Stereological calculations were used to quantify the vascular parameters of the tumours. A comprehensive description of the procedures employed in the present work is given by Weibel (1979) and by Gūnderson (1979). The histological sections were examined at a magnification of 400× by the use of a projecting light microscope. The calculations were based on measurements performed directly on the projections of the sections. A counting frame, 20×20 cm in size, was used for the analysis. To secure a constant reference area equal to the area of the counting frame, and to avoid "edge problems" (Gundersen, 1979), it was taken care that all projections covered the counting frame completely.

As the sections were stained, the necrotic areas could be distinguished from the areas with vital tissue, *i.e.* histologically intact areas. The area fraction of necrotic tissue in each section was determined by point-counting (Weibel, 1979). The volume fraction of necrosis in the tumours ($N$) (Weibel, 1979) was determined by the formula:

$$N = \frac{1}{m} \sum_{i=1}^{m} n_i \qquad (1)$$

where $n_i$ is the area fraction of necrosis in section $i$, and $m$ is the total number of sections.

Due to the contrast medium, the vessels appeared in the projections as dark circles or ellipses, depending on whether they were cut at a right angle or obliquely (Fig. 2). The vessel profiles were classified with respect to vessel diameter. Profiles intersecting the right or the upper edge of the counting frame were registered, whereas profiles intersecting the left or the lower edge were not registered (Gundersen, 1979). Since the section thick-

FIG. 2.—Light micrograph (350×) of E.E. melanoma. Two vessel profiles are seen. The vessel marked A was cut at an oblique angle while the vessel marked B was cut at a right angle.

ness (2 μm) was far less than the vessel diameters, a simple and unbiased registration of the vessel diameters was secured. The smallest diameter was registered on elliptic profiles. Vessels with diameter $<5$ μm were not found in the tumours. The vessel profiles were registered in one of 5 classes, depending on the diameter of the vessels (Table I).

According to Gundersen (1979), vessel length per unit tumour volume ($L_V$) of vessels

TABLE I.—*Classification of vessel profiles*

| Class no. | Diameter range $d_1$–$d_2$ ($\mu$m) | Mean diameter $\bar{d}$ ($\mu$m)* | Mean square diameter $\overline{d^2}$ ($\mu$m$^2$)* |
|---|---|---|---|
| 1 | 5–15 | 10 | 125 |
| 2 | 15–25 | 20 | 425 |
| 3 | 25–35 | 30 | 925 |
| 4 | 35–45 | 40 | 1625 |
| 5† | 45– | 50 | 2525 |

* $\bar{d}=1/2\ (d_1+d_2)$; $\overline{d^2}=1/2\ (d_1{}^2+d_2{}^2)$.

† All vessel profiles with diameter larger than 45 $\mu$m were registered in Class 5. Since the majority of the profiles in this class had diameter $<55\ \mu$m, $\bar{d}=50\ \mu$m and $\overline{d^2}=2525\ \mu$m$^2$ were used in the present calculations.

belonging to the class with mean diameter $\bar{d}i$ was calculated as:

$$L_V\ (\bar{d}) = \frac{2\times(1/m)\sum_{i=1}^{m} Q\ (\bar{d}_i)}{A} \qquad (2)$$

where $Q_i\ (\bar{d})$ is the number of vessel profiles in section $i$ belonging to the class with mean diameter $\bar{d}$, $m$ is the total number of sections, and $A$ is the area of each section (0·25 mm$^2$) which was projected within the counting frame and analysed. Formula (2) is based on two important assumptions. Firstly, isotrophy, which can be obtained by cutting the sections from randomly orientated tumour blocks, as in the present work. Secondly, the thickness of the sections (*e.g.* 2 $\mu$m) must be considerably less than the vessel diameters.

Assuming the vessels to be cylindrical with diameters far less than the vessel length, vessel surface per unit tumour volume ($S_V(\bar{d})$) and vessel volume per unit tumour volume ($V_V(\bar{d})$) (after Gundersen, 1979) were calculated as:

$$S_V(\bar{d}) = \pi\times\bar{d}\times L_V(\bar{d}), \qquad (3)$$

$$V_V(\bar{d}) = \pi/4\times\overline{d^2}\times L_V(\bar{d}). \qquad (4)$$

The vessel profiles were observed almost exclusively in areas with histologically intact tissue and very seldom in necrotic areas. Thus vessel length, surface, and volume per unit histologically intact tumour volume ($L_{V\mathrm{HI}}\ (\bar{d})$, $S_{V\mathrm{HI}}(\bar{d})$, and $V_{V\mathrm{HI}}(\bar{d})$, respectively) were calculated as:

$$L_{V\mathrm{HI}}(\bar{d}) = \frac{L_V\ (\bar{d})}{1-N} \qquad (5)$$

$$S_{V\mathrm{HI}}(\bar{d}) = \pi\times\bar{d}\times L_{V\mathrm{HI}}(\bar{d}) \qquad (6)$$

$$V_{V\mathrm{HI}}(\bar{d}) = \pi/4\times\overline{d^2}\times L_{V\mathrm{HI}}(\bar{d}) \qquad (7)$$

### RESULTS

The fraction of necrosis varied only slightly among individual grafts of the same melanoma. However, a significant variation among the different melanomas was observed. The necrotic fractions were found to be $30\pm1\%$ (G.E.), $32\pm2\%$ (E.E.), $33\pm2\%$ (V.N.), $43\pm4\%$ (M.F.), and $49\pm4\%$ (E.F.).

X-ray images of thick tumour sections indicated that the vascular density varied considerably among the different melanomas. V.N. melanoma was best, while E.F. melanoma was most poorly vascularized (Fig. 3). Vascular structures were usually found only in areas with histologically intact tissue.

Fig. 4 (upper panel) shows histograms for $L_{V\mathrm{HI}}(\bar{d})$ for three different, randomly selected slices from one single tumour of E.E. melanoma. $L_{V\mathrm{HI}}(\bar{d})$ for each tumour slice was calculated as an average of $L_{V\mathrm{HI}}(\bar{d})$ for all blocks of the slice. The number of blocks in each slice is indicated in the Figure. Standard errors (s.e.) are indicated by the vertical bars marked on each column of the histograms. Fig. 4 (lower panel) shows histograms for $L_{V\mathrm{HI}}(\bar{d})$ for three different, randomly selected tumours from different passages of E.E. melanoma. $L_{V\mathrm{HI}}(\bar{d})$ for each tumour was calculated as an average of $L_{V\mathrm{HI}}(\bar{d})$ for all blocks of the tumour. The number of blocks and the s.e. are indicated as in the upper panel. Three main observations were made from Fig. 4. Firstly, the histograms for $L_{V\mathrm{HI}}(\bar{d})$ for the different tumours of E.E. melanoma are not significantly different. Secondly, the histograms for $L_{V\mathrm{HI}}(\bar{d})$ for the different slices from one single tumour of E.E. melanoma are not significantly different. Thirdly, the s.e. are relatively small compared with the mean values for $L_{V\mathrm{HI}}(\bar{d}=10\ \mu\mathrm{m};\ \bar{d}=20\ \mu\mathrm{m})$, *i.e.* the variation in $L_{V\mathrm{HI}}(\bar{d}) = 10\ \mu\mathrm{m};\ \bar{d}=20\ \mu\mathrm{m})$ among the different blocks of a tumour is relatively small. Consequently, the following conclusions were drawn from Fig. 4. (1) The microvascularization of E.E. melanoma in areas with histologically intact tissue does not vary significantly from one part of a

FIG. 3.—X-ray images of 720 μm-thick sections from a well-vascularized (V.N.; left) and a poorly vascularized (E.F.; right) melanoma. Vascular structures are not seen in areas confirmed to be necrotic (indicated by arrows).

tumour to another. In order to observe local variations within a tumour, volumes less than those of the present blocks (3·0–3·5 $mm^3$) have to be analysed. (2) The microvascularization of E.E. melanoma in areas with histologically intact tissue does not vary significantly among individual tumours from different passages.

Similar data and equal conclusions were also obtained for the other 4 melanomas.

Fig. 5 shows histograms for $L_V(\bar{d})$ (upper panel) and $L_{VHI}(\bar{d})$ (lower panel) for all 5. Fig. 6 shows histograms for $S_{VHI}(\bar{d})$ (upper panel) $V_{VHI}(\bar{d})$ (lower panel) for the same melanomas, calculated from the histograms in Fig. 5. Each column of the histograms represents mean values calculated from 4–6 individual tumours, as indicated in the Figures. S.e. are indicated by vertical bars.

Figs 5 and 6 show that $L_{VHI}(\bar{d}=10\ \mu m) > L_{VHI}(\bar{d}=20\ \mu m) > L_{VHI}(\bar{d}=30\ \mu m)$ and that $S_{VHI}(\bar{d}=10\ \mu m) > S_{VHI}(\bar{d}=20\ \mu m) > S_{VHI}(\bar{d}=30\ \mu m)$ for all melanomas, *i.e.* small vessels contribute more to both the total vessel length and the total vessel surface than do larger vessels. The contribution to the total vessel volume from small vessels relative to that from larger ones varies considerably among the different melanomas. Small vessels contribute less than larger vessels in G.E. and M.F. melanomas. On the other hand, in E.F. melanoma the major part of the total vessel volume is due to the smaller vessels. The total vessel volume in E.E. and V.N. melanomas is more evenly distributed among vessels with different diameters.

Fig. 5 (lower panel) shows that $L_{VHI}(\bar{d}=10\ \mu m$; V.N.$) > L_{VHI}(\bar{d}=10\ \mu m$; G.E. and E.E.$) > L_{VHI}(\bar{d}=10\ \mu m$; E.F. and M.F.) and that $L_{VHI}(\bar{d}=20\ \mu m$; V.N. and G.E.$) > L_{VHI}(\bar{d}=20\ \mu m$; E.F. and M.F.). Each of these relations is statistically significant at a level of $P < 0{\cdot}01$. The same relations are valid also for $L_V(\bar{d}=10\ \mu m;\ \bar{d}=20\ \mu m)$ (Fig. 5; upper panel). Since both $S_{VHI}(\bar{d})$ and $V_{VHI}(\bar{d})$ are proportional to $L_{VHI}(\bar{d})$ (Equations (6) and (7)), and $S_V(\bar{d})$ and $V_V(\bar{d})$ are proportional to $L_V(\bar{d})$ (Equations (3) and (4)), the relations quoted above are also valid for $S_{VHI}(\bar{d})$, $V_{VHI}(\bar{d})$, $S_V(\bar{d})$, and $V_V(\bar{d})$. These results demonstrate that the melanomas exhibit individual, characteristic microvascular structures.

The total vessel length, surface, and volume per unit histologically intact tumour volume as well as the mean vessel diameter for the melanomas are presented

FIG. 4.—Upper panel: histograms for $L_{VHI}(d)$ for 3 different, randomly selected slices from one single tumour of E.E. melanoma. Lower panel: histograms for $L_{VHI}(d)$ for 3 different randomly selected tumours of E.E. melanoma. $L_{VHI}(d)$ was calculated as an average of $L_{VHI}(d)$ for the blocks of the slices or the tumours. The number of blocks and the s.e. are indicated in the figure.

in Table II. The values were calculated from the results presented in Figs 5 and 6, as indicated in the text to the Table. The necrotic fractions and the volume-doubling times of the melanomas are also included in Table II. The volume-doubling times were determined by Gomperzian analysis of volumetric growth data, as previously described (Rofstad *et al.*, 1982).

In Fig. 7 the fraction of necrosis is plotted *vs* the total vessel volume per unit histologically intact tumour volume. The Figure indicates that the fraction of necrosis is higher in melanomas with low than with high vascular volumes. Similarly, it can be shown that the fraction of

FIG. 5.—Histograms for $L_V(\bar{d})$ (upper panel) and $L_{VHI}(\bar{d})$ (lower panel) for 5 different melanomas. $L_V(\bar{d})$ and $L_{VHI}(\bar{d})$ were calculated as an average of $L_V(\bar{d})$ and $L_{VHI}(\bar{d})$ for 4–6 individual tumours. The number of tumours and the s.e. are indicated in the figure.

necrosis is probably higher in melanomas with low than with high total vessel length and total vessel surface per unit histologically intact tumour volume. However, with only 5 melanomas statistically significant correlations were not achieved. The $P$ values were in the range 0·05–0·2.

Tumour volume-doubling time is plotted *vs* fraction of necrosis and *vs* total vessel volume per unit histologically intact tumour volume in Fig. 8. The Figure demonstrates that the melanomas with long volume-doubling times have considerably higher necrotic fractions and probably also lower vascular volumes than those with short volume-doubling times. Linear relations were chosen arbitrarily, and thus the curves only indicate a qualitative trend, not an established linearity.

### DISCUSSION

As the vascular bed of human tumour xenografts in nude mice is of murine origin (Giovanella & Fogh, 1978), one may speculate whether the vascular structure is determined by the host animal or by the implanted tumour tissue. The 5 melanoma xenografts studied in the present work showed individual, characteristic vascular structures. Both the histograms for $L_{VHI}(\bar{d})$ and $L_V(\bar{d})$ and hence those for $S_{VHI}(\bar{d})$, $V_{VHI}(\bar{d})$, $S_V(\bar{d})$, and $V_V(\bar{d})$ (Figs 5 and 6), the total vessel length, surface, and volume per unit histologically intact tumour volume (Table II), and the

FIG. 6.—Histograms for $S_{VHI}(\bar{d})$ (upper panel) and $V_{VHI}(\bar{d})$ (lower panel) for 5 different melanomas. $S_{VHI}(\bar{d})$ and $V_{VHI}(\bar{d})$ were calculated as an average of $S_{VHI}(\bar{d})$ and $V_{VHI}(\bar{d})$ for 4–6 individual tumours. The number of tumours and the s.e. are indicated in the figure.

mean vessel diameter (Table II) varied significantly among the different melanomas. Since the melanomas were implanted at the same site in nude mice, the present study indicates that the vascular structure of human tumour xenografts is, at least in part, determined by the human parenchymal tumour cells, possibly by the ability of the cells to synthesize and secrete tumour-angiogenesis factor (Folkman, 1976).

The melanomas also showed individual, characteristic necrotic fractions. The data indicated that the necrotic fractions were higher in poorly than in well vascularized melanomas (Fig. 7). This observation was expected since the supply of oxygen and nutrients as well as the removal of necrotic

TABLE II.—*Mean vessel diameter* ($\bar{D}$), *necrotic fraction* ($N$), *tumour volume-doubling time* ($T_d$), *and total vessel length* ($L_{VHI}$), *surface* ($S_{VHI}$), *and volume* ($V_{VHI}$) *per unit histologically intact tumour volume for human melanoma xenografts*

| Melanoma | $L_{VHI}$ ± s.e.* (mm/mm³) | $S_{VHI}$ ± s.e.* (mm²/mm³) | $V_{VHI}$ ± s.e.* (mm³/mm³) | $\bar{D}$ ± s.e.* (μm) | $N$ ± s.e. (%) | $T_d$† (days) |
|---|---|---|---|---|---|---|
| V.N. | 80 ± 4 | 3·8 ± 0·2 | 0·022 ± 0·002 | 15·0 ± 0·4 | 33 ± 2 | 6·2 |
| G.E. | 55 ± 1 | 3·1 ± 0·1 | 0·021 ± 0·001 | 18·1 ± 0·4 | 30 ± 1 | 4·2 |
| E.F. | 32 ± 2 | 1·6 ± 0·1 | 0·009 ± 0·001 | 16·0 ± 0·3 | 49 ± 4 | 21·6 |
| E.E. | 46 ± 2 | 2·5 ± 0·2 | 0·015 ± 0·002 | 16·9 ± 0·5 | 32 ± 2 | 4·4 |
| M.F. | 36 ± 2 | 2·2 ± 0·1 | 0·015 ± 0·001 | 18·8 ± 0·5 | 43 ± 4 | 20·0 |

* $L_{VHI} = \sum_{i=1}^{5} L_{VHI}(\bar{d}_i)$; $S_{VHI} = \sum_{i=1}^{5} S_{VHI}(\bar{d}_i)$; $V_{VHI} = \sum_{i=1}^{5} V_{VHI}(\bar{d}_i)$; $\bar{D} = \dfrac{\sum_{i=1}^{5} \bar{d}_i L_{VHI}(\bar{d}_i)}{\sum_{i=1}^{5} L_{VHI}(\bar{d}_i)}$

† $T_d$ was determined for tumour volumes of about 200 mm³ as described previously (Rofstad *et al.*, 1982).

FIG. 7.—Volume fraction of necrotic tissue *vs* total vessel volume per unit histologically intact tumour volume (Table II) for human melanoma xenografts. Each point represents mean values and s.e. calculated from 4–6 individual tumours.

tissue take place *via* the vascular system. However, other factors than the vascularization, such as oxygen tension, cell respiration, cell density, diffusion resistance, and extravascular streaming may also vary considerably among different tumours (Cassarett, 1974) and hence affect the necrotic fractions. The present results on human melanoma xenografts are in agreement with those reported by Falk (1978), who studied 4 rat sarcomas and showed that hypovascular tumours had relatively high necrotic fractions compared with better vascularized tumours.

The melanomas with long volume-doubling times (E.F. and M.F.) had higher necrotic fractions (Fig. 8(*a*)) and probably also lower vessel volume per unit histologically intact tumour volume (Fig. 8(*b*)) than those with short volume-doubling times (E.E., G.E., and V.N.). Thus the rate of volume growth of the melanomas may be limited, at least in part, by the ability of the tumour tissue to develop its vascular system. Previous investigations have indicated that the melanomas with long volume-doubling times have both lower growth fractions and higher cell-loss factors than those with short volume-doubling times (Rofstad *et al.*, 1982). Consequently, inadequate supply of oxygen and nutrients due to poor vascularization may result in low growth fractions and high cell-loss factors.

The total vessel volume of the melanoma xenografts ranged from 0·9 to 2·2% of the histologically intact tumour volume (Table II). These values are significantly

FIG. 8.—Tumour volume-doubling time *vs* (a) volume fraction of necrotic tissue and (b) total vessel volume per unit histologically intact tumour volume (Table II) for human melanoma xenografts. Each point represents mean values and s.e. calculated from 4–6 individual tumours.

lower than those reported for many rodent tumours grown in syngeneic hosts. The vascular fraction was reported to be about 17% for the murine C3H/Bi mammary carcinoma (Hilmas & Gillette, 1975), 15–18% for the murine 72j mammary adenocarcinoma (Vogel, 1965), about 9% for the murine SCK mammary carcinoma (Song *et al.*, 1980), and 3–15% for the rat AH109A hepatoma (Yamaura & Matzusawa, 1979). However, the difference between the vascular volumes measured for these rodent tumours and for the present melanoma xenografts may to some extent be due to the use of different experimental techniques. The mean vessel diameters for the melanomas ranged from $15{\cdot}0 \pm 0{\cdot}4$ to $18{\cdot}8 \pm 0{\cdot}5$ $\mu$m (Table II), while the C3H/Bi mammary carcinoma was reported to have a mean vessel diameter of 27 $\mu$m at a tumour volume of about 200 $mm^3$ (Hilmas & Gillette, 1975). Consequently, a larger part of the vascular system is probably due to small vessels in the melanoma xenografts than in the C3H/Bi mammary carcinoma, indicating an efficient utilization of the total blood flow in the xenografts.

Instruction in stereology by Dr A. Reith and Dr H. J. Gundersen at the 5th Scandinavian Course in Stereology is gratefully acknowledged. Dr J. Sokolowski and Mr A. Rønnestad are thanked for their valuable advice on the preparation and injection of the contrast medium. The staff at the Laboratory for Histology, Department of Pathology, is thanked for excellent technical assistance. Financial support from The Norwegian Cancer Society, The Norwegian Research Council for Science and the Humanities, and The Nansen Scientific Fund is gratefully acknowledged.

### REFERENCES

CASSARETT, G. W. (1974) Importance of vascularity, tumour bed and other general aspects of radiosensitivity. In *The Biological and Clinical Basis of Radiosensitivity*, (Ed. Friedman). Springfield: Charles C. Thomas. p. 181.

DEWEY, W. C., FREEMAN, M. L., RAAPHORST, G. P. & 7 others (1980) Cell biology of hyperthermia and radiation. In *Radiation Biology in Cancer Research* (Ed. Meyn & Withers). New York: Raven Press. p. 589.

FALK, P. (1978) Patterns of vasculature in two pairs of related fibrosarcomas in the rat and their relation to tumour responses to single large doses of radiation. *Eur. J. Cancer*, **14**, 237.

FIELD, S. B. & BLEEHEN, N. M. (1979) Hyperthermia in the treatment of cancer. *Cancer Treat. Rev.*, **6**, 63.

FOLKMAN, J. (1976) The vascularization of tumors. *Sci. Am.*, **234**, 58.

GIOVANELLA, B. C. & FOGH, J. (1978) Present and future trends in investigations with the nude mouse as a recipient of human tumor transplants. In *The Nude Mouse in Experimental and Clinical Research* (Ed. Fogh & Giovanella). New York: Academic Press. p. 281.

GUNDERSEN, H. J. (1979) Estimations of tubuli or cylinder $L_V$, $S_V$, and $V_V$ on thick sections. *J. Microsc.*, **117**, 333.

HALL, E. J. (1978) *Radiobiology for the Radiologist*. New York: Harper & Row.

HILMAS, D. E. & GILLETTE, E. L. (1975) Microvasculature of C3H/Bi mouse mammary tumors after X-irradiation. *Radiat. Res.*, **61**, 128.

KENNEDY, K. A., TEICHER, B. A., ROCKWELL, S. & SARTORELLI, A. C. (1980) The hypoxic tumor cell: A target for selective cancer chemotherapy. *Biochem. Pharmacol.*, **29**, 1.

POWERS, W. E. & TOLMACH, L. J. (1963) A multicomponent X-ray survival curve for mouse lymphosarcoma cells irradiated *in vivo*. *Nature*, **197**, 710.

ROFSTAD, E. K. & BRUSTAD, T. (1981) Radiation response *in vitro* of cells from five human malignant melanoma xenografts. *Int. J. Radiat. Biol.*, **40**, 677.

ROFSTAD, E. K. & BRUSTAD, T. (1982) Effect of hyperthermia on human melanoma cells heated either as solid tumors in athymic nude mice or *in vitro*. *Cancer* (in press).

ROFSTAD, E. K., FODSTAD, Ø. & LINDMO, T. (1982) Growth characteristics of human melanoma xenografts. *Cell Tissue Kinet.* (in press).

SONG, C. W., KANG, M. S., RHEE, J. G. & LEVITT, S. H. (1980) Vascular damage and delayed cell death in tumours after hyperthermia. *Br. J. Cancer*, **41**, 309.

STEEL, G. G. (1978) The growth and therapeutic response of human tumours in immune deficient mice. *Bull. Cancer*, **65**, 465.

STEEL, G. G. & PECKHAM, M. J. (1980) Human tumour xenografts: A critical appraisal. *Br. J. Cancer*, **41** (Suppl. IV), 133.

TANNOCK, I. F. (1972) Oxygen diffusion and the distribution of cellular radiosensitivity in tumours. *Br. J. Radiol.*, **45**, 515.

THOMLINSON, R. H. & GRAY, L. H. (1955) The histological structure of some human lung cancers and the possible implications for radiotherapy. *Br. J. Cancer*, **9**, 539.

VOGEL, A. W. (1965) Intratumoral vascular changes with increased size of a mammary adenocarcinoma: New methods and results. *J. Natl Cancer Inst.*, **34**, 571.

WEIBEL, E. R. (1979) *Stereological Methods*. Vol. I. *Practical Methods*. New York: Academic Press.

YAMAURA, H. & MATSUZAWA, T. (1979) Tumour regrowth after irradiation. An experimental approach. *Int. J. Radiat. Biol.*, **35**, 201.